# Non-invasive treatment of patients with upper extremity spasticity following stroke using paired trans-spinal and peripheral direct current stimulation

**DOI:** 10.1186/s42234-019-0028-9

**Published:** 2019-07-23

**Authors:** Alexandra Paget-Blanc, Johanna L. Chang, Maira Saul, Regina Lin, Zaghloul Ahmed, Bruce T. Volpe

**Affiliations:** 1 Feinstein Institute for Medical Research, Biomedical Science Division, Biomedical Sciences /Robot Lab, Laboratory of Clinical Neurorehabilitation Research, 350 Community Dr, Manhasset, NY 11030 USA; 2 BARC Global Central Laboratory, 5 Delaware Dr, Hyde Park, NY 11042 USA; 3 College of Staten Island, Department of Physical Therapy, Center for Developmental Neuroscience, Staten Island, NY 10314 USA; 4 Graduate Center, City University of New York, New York, NY USA

**Keywords:** Stroke, Spasticity, tsDCS+ pDCS, Trans-spinal direct current stimulation, Paired spinal and peripheral stimulation, Upper motor neuron syndrome, CVA, Hemiparesis, Motor rehabilitation

## Abstract

**Background:**

Muscle spasticity is a common impediment to motor recovery in patients with chronic stroke. Standard-of-care treatments such as botulinum toxin injections can temporarily relieve muscle stiffness and pain associated with spasticity, but often at the expense of increased muscle weakness. Recent preclinical investigations of a non-invasive treatment that pairs trans-spinal direct current stimulation and peripheral nerve direct current stimulation (tsDCS+pDCS) provided promising data for a novel approach based on bioelectronic medicine for the treatment of patients with post-stroke spasticity.

**Methods:**

Twenty-six patients with upper limb hemiparesis and wrist spasticity at least 6 months after their initial stroke participated in this single-blind crossover design study to test whether tsDCS+pDCS reduces chronic upper-extremity spasticity. Subjects received five consecutive daily sessions (20 min of stimulation or sham) of anodal tsDCS+pDCS, separated by a one-week washout period. The sham condition always preceded the active condition. Clinical and objective measures of spasticity and motor function were collected before and after each condition, and for five weeks after the completion of the active intervention.

**Results:**

Subjects treated with active tsDCS+pDCS demonstrated significant reductions in both Modified Tardieu Scale scores (summed across the upper limb, *P < 0.05*), and in objective torque measures (Nm) of the spastic catch response at the wrist flexor (*P < 0.05),* compared to the sham condition*.* Motor function also improved significantly (measured by the Fugl-Meyer and Wolf Motor Function Test; *P < 0.*05 for both tests) after active treatment.

**Conclusions:**

tsDCS+pDCS intervention alone significantly reduced upper limb spasticity in participants with stroke. Decreased spasticity was persistent for five weeks after treatment, and was accompanied by improved motor function even though patients were unsupervised and there was no prescribed activity or training during that interval.

**Trial registration:**

NCT03080454, March 15, 2017.

## Background

Spasticity is a motor disorder that occurs in 20–43% of individuals after stroke, and is defined as a velocity dependent increase in the tonic stretch reflex resulting from simultaneous co-contraction of agonist and antagonist muscles that often leads to increased stiffness and permanent contracture. Spasticity is one of a constellation of positive and negative symptoms experienced as a result of upper motor neuron lesions after stroke, and also includes weakness, loss of dexterity, decreased motor control and reduced endurance (Sunnerhagen, 2016; Sommerfeld et al., 2012; Levy et al., 2018; Lundström et al., 2010; Lance, 1980). Direct costs for stroke survivors with spasticity are four times greater than for those without spasticity during the first 12 months after cerebral infarct (Lundström et al., 2010), indicating that a significant reduction in healthcare expenditures could be achieved for these patients if spasticity could be better controlled. Spasticity occurs more frequently in the upper rather than the lower limbs, and this regional propensity contributes to less recovery of upper limb independence, often resulting in near permanent impairment of the wrist and hand (Sommerfeld et al., 2012). Some investigators have demonstrated that effective upper limb rehabilitation requires intensive, repetitive, activity dependent learning (Chang et al., 2017; Volpe et al., 2009; Lo et al., 2010). However, despite aggressive therapy, residual spasticity frequently inhibits active wrist and finger extension, prohibiting any attainment of new functional capacity. When the spasticity continues to worsen and becomes severe or causes pain, the standard-of-care is botulinum toxin injection (Levy et al., 2018).

Trans-spinal direct current stimulation (tsDCS) has a long history as a procedure to modulate spinal cord activity (Eccles et al., 1962), and recent work has demonstrated normalization of muscle tone with improved motor function. Pre-clinical experiments (Ahmed, 2011; Ahmed, 2013a; Ahmed, 2013b; Ahmed, 2014; Wieraszko & Ahmed, 2016) show that the electric fields induce differential polarization of the spinal motor neurons so that a dendritic Ca^2+^ persistent inward current modulates motor neuron excitability. More specifically, trans-spinal direct current stimulation combined with peripheral direct current stimulation (tsDCS + pDCS) produced polarity dependent changes in muscle tone, such that cathodal stimulation (flowing from the periphery to the spinal cord) increased muscle tone, and anodal stimulation (flowing from the spinal cord to the periphery) decreased muscle tone (Ahmed, 2014). Furthermore, in healthy volunteers, investigators demonstrated that 2.5 mA of tsDCS altered the H-reflex response such that 20 min of anodal tsDCS significantly reduced H-reflex post-activation depression (e.g. facilitated H-reflex) and cathodal tsDCS increased post-activation depression (e.g. decreased H-reflex) (Winkler et al., 2010). A similar report also in healthy volunteers demonstrated that cathodal tsDCS to the cervical or lower thoracic spinal cord significantly improved motor recruitment recorded at the ulnar and medial nerves with incremental multipoint stimulation of the abductor digiti minimi (ADM) and the abductor pollicis brevis (APB) (Bocci et al., 2014). In a recent, small (11 subjects) study of patients with hereditary spastic paraplegia, investigators demonstrated that five twice-daily sessions of 2.0 mA anodal tsDCS to the thoracic spinal cord significantly decreased Modified Ashworth Scale measures of lower limb spasticity for as long as 2 months following the end of stimulation, but no significant functional improvements were observed (Ardolino et al., 2018).

Taken together, these data suggest that tsDCS acts to modulate spinal motor responses resulting in positive therapeutic effects, and combining tsDCS with pDCS may enhance these effects. In this single-blind cross-over design study, we investigated whether 5 consecutive days of 20-min active, paired tsDCS+pDCS would reduce upper limb muscle spasticity and improve upper limb functional use in patients with chronic stroke as compared to an equivalent sham condition.

## Methods

### Participants

Twenty-six subjects with chronic (> 6 months) upper limb hemiparesis and spasticity of the wrist and hand who met the eligibility criteria were recruited by the physicians in the departments of Neurology and Physical Medicine and Rehabilitation at Northwell Health and enrolled for the study. Inclusion criteria were: a) ≥ 18 years of age; b) first, single unilateral lesion with diagnosis verified by brain imaging (CT or MRI) that occurred at least 6 months prior; c) cognitive function sufficient to understand the experiments and follow instructions; d) a Modified Tardieu Scale score between 1 and 4 points for wrist flexors and/or extensors; e) a minimum of 15 degrees wrist passive ROM for wrist flexion and extension from wrist neutral position. Exclusion criteria were: a) focal brainstem or thalamic infarcts; b) prior surgical treatments for spasticity of the upper limb; c) ongoing use of CNS-active medication; d) ongoing use of psychoactive medications, such as stimulants, antidepressants, and anti-psychotic medications; e) botulinum toxin or phenol alcohol treatment within 12 weeks of enrollment; f) pregnancy in women, as determined by self-report; g) history of spinal cord injury or weakness; h) chronic pain, defined by a report of a “5” or greater on the Wong-Baker Pain Scale (Garra et al., 2010) i) peripheral neuropathy including insulin dependent diabetes as determined by case history; j) presence of additional tsDCS risk factors including damaged skin at any of the stimulation sites, presence of implantable devices, highly conductive metal in any part of the body, and seizure history in the past 36 months; k) missing more than 2 sham or treatment sessions. A preliminary safety trial, using comparable selection criteria and stimulation parameters, demonstrated that a single 20-min session of tsDCS+pDCS, caused no adverse effects in eleven subjects and six healthy controls.

A total of 387 patients were screened for the study, resulting in 73 (19%) subjects who were eligible for enrollment. Twenty-six subjects ultimately enrolled. Two subjects dropped out after consent but before receiving treatment due to unrelated health events, and one subject missed measures and treatments during the sham condition and thus were excluded from the analysis. Twenty-three subjects completed sham and active treatment conditions. However, three subjects were significant outliers (defined as >2SDs from mean change for objective measures; objective measures were most susceptible to instrumental noise) and therefore were excluded from the statistical analysis. Consequently, a total of 20 participants received both treatment conditions and were included in the primary analysis comparing treatment effects between active and sham immediately following the 5th treatment and at one-week follow-up. To analyze the robustness of a treatment effect (long-term effect), 16 patients were included; four patients missed more than two consecutives follow up sessions and were excluded. The demographic characteristics are reported in Table 1. All visits were conducted in the clinical robotics and non-invasive brain stimulation suite at the Feinstein Institute for Medical Research.

**Table 1:** Patients’ demographics

Parameters (*n* = 26)	Mean (SEM)	Range
Sex, F/M, n	10/16	N/A
Age	63.64 (2.58)	26–83
Time after stroke, years	3.04 (0.89)	0.5–21
Type of stroke (Ischemic/Hemorrhagic), n	22/4	N/A
Affected side (Dominant/Nondominant), n	12/14	N/A
Baseline Fugl-Meyer	27.61 (3.02)	8–55
Baseline MTS upper extremity	23.81 (1.13)	13.67–36.0
Baseline MTS flexor	2.75 (0.18)	1.50–4

### Study design

This was a single-blind cross over design study in which subjects were told they would receive both active and sham stimulation conditions, but were not told the order of stimulation. All subjects acclimated to the procedures in two to three baseline visits prior to treatment during which instrumental and clinical assessments were performed. After the lead-in period, subjects received five consecutive, 20-min sessions of sham stimulation (visits 1–5) followed by a week wash-out period and then another five consecutive 20-min sessions of anodal tsDCS+pDCS (visits 7–11). Subjects attended a one-week follow-up evaluation after the end of sham condition (visit 6) and five weekly follow-ups after the end of active condition (visits 12–16). Sham stimulation always preceded the active condition in order to measure the potential long term effects of anodal tsDCS+pDCS. Time-matched, long term follow-ups in the sham condition were not feasible due to the excess burden required for study subjects to return for multiple follow-up visits. Clinical and objective measures were collected before the first session, immediately following the last session of each condition, and at each subsequent follow-up evaluation.

**Fig. 1:**
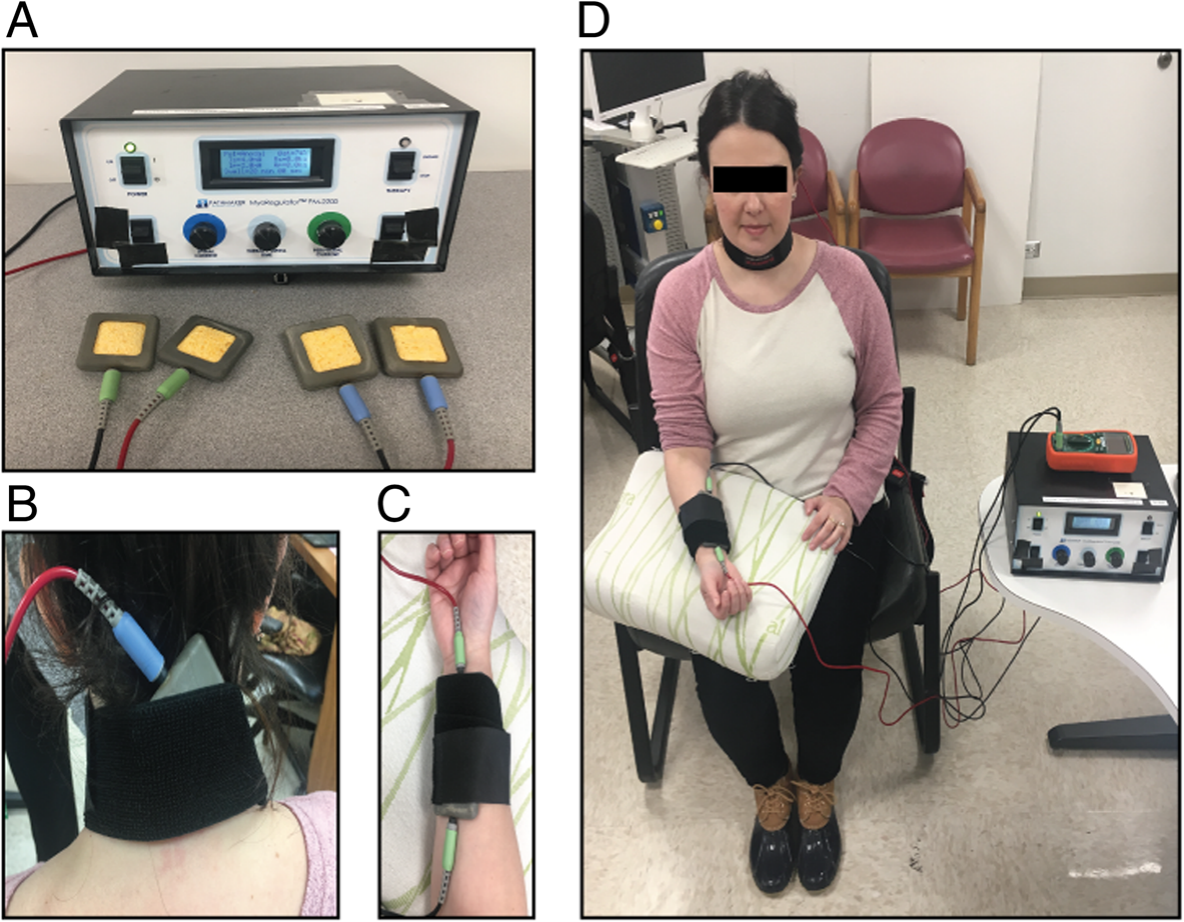
tsDCS+pDCS treatment delivered by the MyoRegulator®paired trans-spinal direct current stimulation with peripheral direct current stimulation. **a** PathMaker MyoRegulator® control panel with two pairs of 2x2in sponge-electrodes. **b** Anode electrode placed on the spine at C6 level for spinal stimulation (4 mA); and cathode electrode placed on the abdomen. **c** Second set of electrodes, anode and cathode positioned on the median nerve. **d** Subject receiving tsDCS+pDCS treatment: 4 mA for spinal stimulation and 1 mA for peripheral nerve stimulation

tsDCS+pDCS treatment was delivered by a patented device called the MyoRegulator® in development by PathMaker Neurosystems Inc. (Boston, Massachusetts). MyoRegulator is an investigational microprocessor-controlled device powered by rechargeable batteries that delivers non-invasive, paired tsDCS+pDCS stimulation (trademarked as DoubleStim™) using two pairs of 2x2in sponge-electrodes as shown in Fig. 1. Each sponge electrode was soaked with saline (0.9% NaCl) prior to application. The first set of electrodes delivered up to 4 mA of trans-spinal direct current (tsDCS), with the anode placed on the spine at the C6 level, and the cathode was placed above the iliac crest on the abdomen. For the second set of electrodes, the anode was placed proximal to cathode on the median nerve for peripheral nerve direct current stimulation (pDCS) at 1 mA. Spinal current was initially set at 2.5 mA for approximately 1 min to confirm that the stimulation was tolerable, after which the current was progressively increased to 3 mA for 1 min then to 4 mA. This thresholding procedure occurred prior to every session. All subjects, except one during a single session, tolerated 4 mA. For both the active and sham conditions, the MyoRegulator® device was designed to gradually ramp the currents to the set current level at 0.1 mA/sec for patient comfort. When delivering treatment in sham mode, the device was designed to hold at the set current for 5 s, then ramp the current down to zero at 0.1 mA/sec. At the end of the set treatment time (20 min), the currents again ramped up to the set current levels at 0.1 mA/sec, were held for 5 s, then ramped down to zero. This sham treatment regimen provided the patient with the sensation of receiving treatment without providing sufficient stimulation for any therapeutic effect (Gandiga et al., 2006; Brunoni et al., 2014).

### Instrumental assessment of spasticity

Passive muscle resistance was measured using a calibrated force transducer driven by a computer-controlled stepper motor, which registered torque (Nm) during passive stretching of the wrist at slow and fast velocities (Ahmed, 2011; Ahmed, 2013a; Ahmed, 2013b; Ahmed, 2014). The device was calibrated using a one pound weight, that hung freely from the hand grip, and then with the motor on hold, the torque sensor output was zeroed. The voltage output that resulted from the one pound weight was then recorded and used to convert voltage signal to weight (LabChart; ADIinstruments). We measured the length of the movement arm and the distance travelled to generate the torque. Then the device was placed on a height-adjustable table that could be positioned to accommodate the impaired limb. Table height and chair position were recorded and reproducibly adjusted for each patient so that the subject’s forearm rested comfortably on the device, at neutral position, with their elbow flexed at 90° and shoulder abducted at less than 90°. The patient’s hand was strapped to a joystick which moved the wrist passively, while the forearm was stabilized on the static part of the device with two additional straps. Range of motion (ROM) for wrist extension was determined at slow speed followed by fast speed (27.1 degrees/s, and 144.4 degrees/s respectively). At each velocity, the subject’s hand was passively moved 3–4 times with a 3–5 s interval between each movement. Peak torque was calculated for both slow and fast velocities from the slope of the force-time curve using a customized routine of LabChart software (ADInstruments); and the mean torque was calculated from the integral of the slope. The peak torque reflected the ‘clasp-knife’ spastic response (equivalent of a score of ‘2’on Modified Tardieu Scale) whereas the mean torque characterized both the spastic catch and generalized muscle stiffness. Because most patients had both spasticity and muscle stiffness, we determined that the mean torque was a more reliable and consistent parameter, and therefore it was used for all objective statistical analyses. A surface electromyography (sEMG) electrode was placed on the FCR muscle to monitor muscle activity. Data were collected (PowerLab System, ADInstruments, Oxford, UK), at a sample rate of 10 KHz, and filtered at a low pass of 2 kHz and a high pass of 100 Hz and save for offline analysis.

### Clinical assessments

#### Modified Tardieu scale (MTS)

The Modified Tardieu Scale (MTS) is a validated and reliable measure of spasticity that accounts for resistance to passive movement at both slow (V1) and fast speeds (V2) based on a 6-point ordinal scale (0-*no resistance*, to 5-*joint immobile*)*.* (Singh et al., 2011; Paulis et al., 2011). A recent publication used another standardized clinical measure of spasticity, the Modified Ashworth Scale (MAS), and evaluated summed scores across upper extremity muscle groups to reflect a primary outcome measure (Pundik et al., 2014). Based on this precedent, and the expectation that we might see improvement in multiple muscles along the path of current flow, we utilized summed scores for the MTS across the upper extremity muscles (MTS_UE_), as the MTS has been demonstrated to be more sensitive to changes in spasticity as compared to the MAS (Haugh et al., 2006; Akpinar et al., 2017; Mehrholz et al., 2005). For the MTE_UE_, we summed the measurements across the shoulder (horizontal adductors, vertical adductors and internal rotators), elbow (flexors, extensors, pronators and supinators) and wrist/hand (flexors, extensors, fingers and palmar interossei+ flexor digitorum superficialis). MTS score at the wrist flexor alone (MTS_flexor_) was also collected to have a direct clinical comparison with the objective measures. Based on previous MAS studies examining a single joint, we defined a 1-point reduction in MTS_flexor_ as the minimal clinically significant improvement (MCID) (Brashear et al., 2002). As neither the MAS nor the MTS have a documented MCID for summed measurements, we defined a response to treatment as at least a 3-point reduction in MTS_UE_.

#### Upper-extremity Fugl-Meyer (UE-FM)

The Upper-Extremity Fugl-Meyer (UE-FM) scale is a validated and reliable instrument for evaluation of performance-based impairment in patients with hemiparesis after stroke. Each item is scored on a 3-point ordinal scale (0 = *cannot perform*, 1 = *performs partially*, 2 = *performs fully*) with a possible maximum score of 66 (Gladstone et al., 2002; Hsieh et al., 2009; Kim et al., 2012). The MDC (Minimum Detectable Change) and MCID for UE-FM are 1.56 points and 4.25 points, respectively (Page et al., 2012; Toluee Achacheluee et al., 2016).

#### Wolf Motor function

The Wolf Motor Function Test is a validated and reliable instrument for assessment of upper extremity function consisting of fifteen (15) motor-based tasks and two (2) strength-based tasks. The speed at which each functional task is completed is measured by performance time, (120 s maximum; WMFT Time); and the quality of the movement during each task is assessed with the Functional Ability Scale (FAS). For FAS, items are scored on a 6-point ordinal scale, which ranges from 0 (*does not attempt with the involved arm),* to 5 (*arm does participate/movement appears to be normal)* (Hsieh et al., 2009; Wolf et al., 2001; Hodics et al., 2012). The total WMFT Time and FAS for the 15 timed-tasks were determined, with scores ranging from 15 to 18,000 s for WMFT Time and 0–75 points for FAS; and percent change from baseline was calculated for both measurements. Photos and/or video recordings were taken during this assessment for review and scoring by approved clinicians with patient consent.

### Statistical analysis

**Fig. 2:**
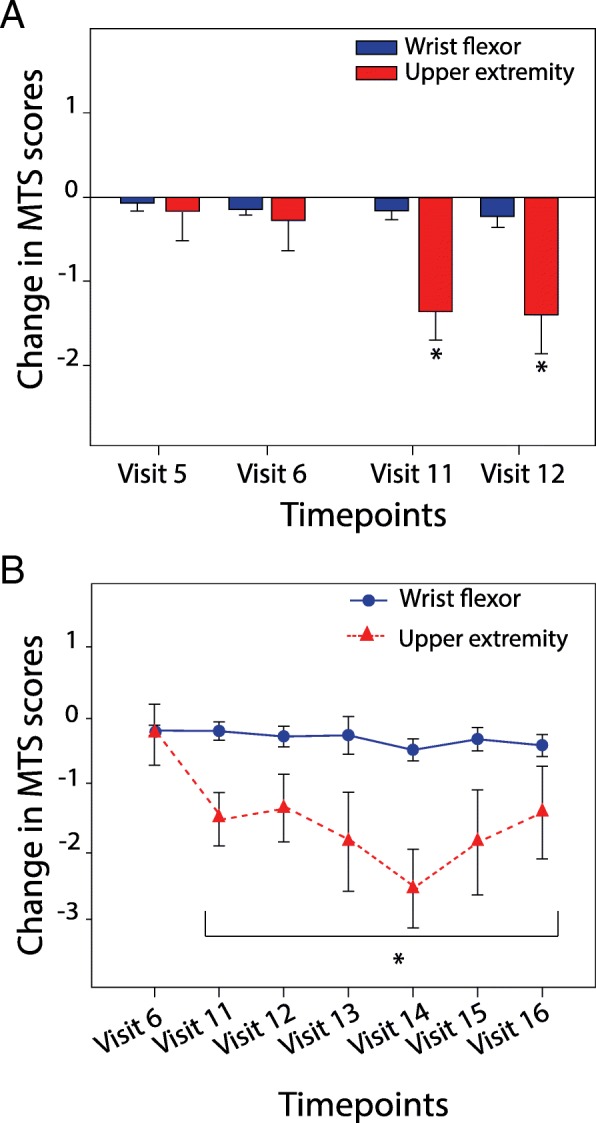
tsDCS+pDCS acutely and chronically reduced clinically measured spasticity. **a** MTS score change (mean +/− SEM) immediately following 5 days of sham treatment and one week later (visits 5,6); 5 days of active treatment and one week later (visits 11,12; *P < 0.05, ANOVA). **b** MTS score change (mean+/− SEM) after active treatment measured weekly for five follow up visits (11–16; *P < 0.05, ANOVA, visit 6 control)

For analysis of the clinical and objective measures, the change from baseline was calculated for each condition at end of sham or treatment (visit 5 and 11 respectively, see Fig. 2), and also at each study follow-up (visit 6 for sham and visit 12–16 for active treatment). Baseline measures for the active treatment occurred one to three days prior to treatment (visit 6, 7). To test the robustness of any active treatment effect over the 5 week follow-up period, we used the measure at the start of the active treatment period subtracted from the measurements obtained at visit 12–16 (weeks 2 through 5) of the follow up period. Results are presented as mean ± standard error of the mean (SEM) and are provided for the change in MTS (MTS_UE_ and MTS_flexor_), change in the secondary motor outcome scores (Fugl-Meyer and Wolf Motor Function Test (WMFT-FAS and WMFT-Time), and percent change in the objective measures. Normal distribution and homogeneity of the parameters were verified using Shapiro-Wilk procedure. Among the parameters evaluated, torque data and MTS_UE_ followed normal distribution and were therefore analyzed using parametric statistics. Acute effects of tsDCS+pDCS on these two parameters were evaluated separately using a two-way repeated-measure analysis of variance (RM-ANOVA) with condition and time as independent variables and change from baseline as the dependent variable. A one-way repeated-measures ANOVA was used to evaluate the long term effects of active tsDCS+pDCS. If the ANOVA was significant at the 0.05 level, a *post-hoc* analysis using Holm-Sidak pairwise comparison was performed. Because the Friedman ANOVA does not allow for any missing values, nonparametric data were analyzed using Wilcoxon signed-rank tests and Bonferroni corrections were applied to adjust for multiple comparisons, with a critical significant level set a .00625 or lower (.05/8). All statistical analysis was performed using SigmaPlot12.5.

## Results

### Clinical spasticity outcomes: modified Tardieu scale

**Fig. 3:**
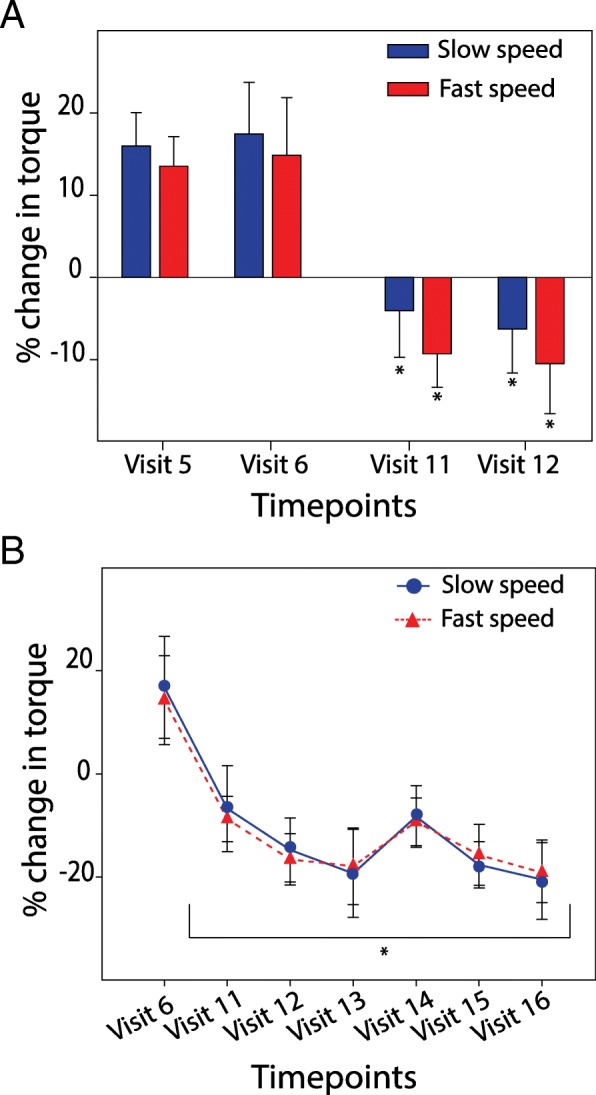
tsDCS+pDCS acutely and chronically reduced objectively measured spasticity. **a** Change in torque resistance (mean percent +/− SEM) immediately following 5 days of sham treatment and one week later (visits 5,6); 5 days of active treatment and one week later (visits 11,12; *P < 0.05, ANOVA). **b** Change in torque resistance (mean percent +/− SEM) after active treatment measured weekly for five follow-up visits (11–16; *P < 0.05, ANOVA, visit 6 control)

For MTS_UE_, the sum of the scores across 11 joints of the upper-extremity, analysis of the acute effects of treatment revealed a significant main effect of condition (F_1,19_ = 13.88; *P* = .001, ANOVA) and post hoc analysis showed significant differences at visit 11 and 12 compared to visit 5 and 6 (*P* = .002 and *P* = .019, respectively, ANOVA; Fig. 2a). Analysis of the durability of the treatment (from visit 7 to visit 16) revealed that MTS_UE_ scores were significantly decreased over time (F_1,15_ = 3.70; *P* = .005, ANOVA; Fig. 3a). And post hoc analysis showed significant differences at every time point, using visit 6 as control (*P* < .05). Maximal decrease in MTS_UE_ occurred at visit 14 (3 weeks after treatment) with a mean change of − 2.53 ± 0.58 points. For MTS_flexor_, paired-sample Wilcoxon Signed Rank revealed no significant difference in scores after sham and active treatment. Although not significant, maximal change in MTS_flexor_ was noted at visit 14 with a mean change of − 0.47 ± 0.17 points (*P* = .031). Using a 1-point change in MTS_flexor_ and 3-point change in MTS_UE_ as clinically significant, the responder rates are 43.75% (7/16) and 56.3% (9/16) respectively. Raw scores a presented Table S1 the in supplemental.

### Objective spasticity outcomes: muscle resistance

Analysis of the acute effects of treatment revealed a significant main effect of condition on percent change in muscle resistance at slow and fast speeds (F_1,19_ = 10.58, *P* = .004; F_1,19_ = 12.40, *P* = .002, respectively; ANOVA). At both speeds post hoc analysis revealed significant difference at visit 11 and 12 compared to visit 5 and 6, respectively (slow: *P* = .030 and fast *P* = .010, for comparison of visit 11 and 5; slow *P* = .008 and fast *P* = .011 for comparison of visit 12 and 6). While muscle resistance tested at visit 11(immediately after active treatment) decreased by 6 and 9% (for slow and fast speeds, respectively), at visit 5 (after sham treatment) muscle resistance increased by 13 and 17% (for fast and slow speeds, respectively; Fig. 3a). Further analysis to test for durability of the alteration in spasticity revealed significant difference in percent change in muscle resistance after active treatment, visit 11–16, at both slow and fast speeds (F_6,15_ = 3.55, *P* = .004; F_6,15_ = 3.72, *P* = .003, respectively, ANOVA; Fig. 3b). Holm-Sidak pairwise comparison revealed significant differences in both slow and fast speeds (*P* < .02) at each visit subsequent to the end of active treatment (visit 11–16). Optimal response was noted at visit 13, with a mean decrease of − 20.53 ± 7.69% at slow speed and − 19.20 ± 5.83% at fast speed. (Fig. 3b). The persistent and robust treatment effects on spasticity were consistent across both the clinical and objective measurements. Raw values are presented in Table S2 in the supplemental.

### Clinical outcomes for motor function

**Fig. 4:**
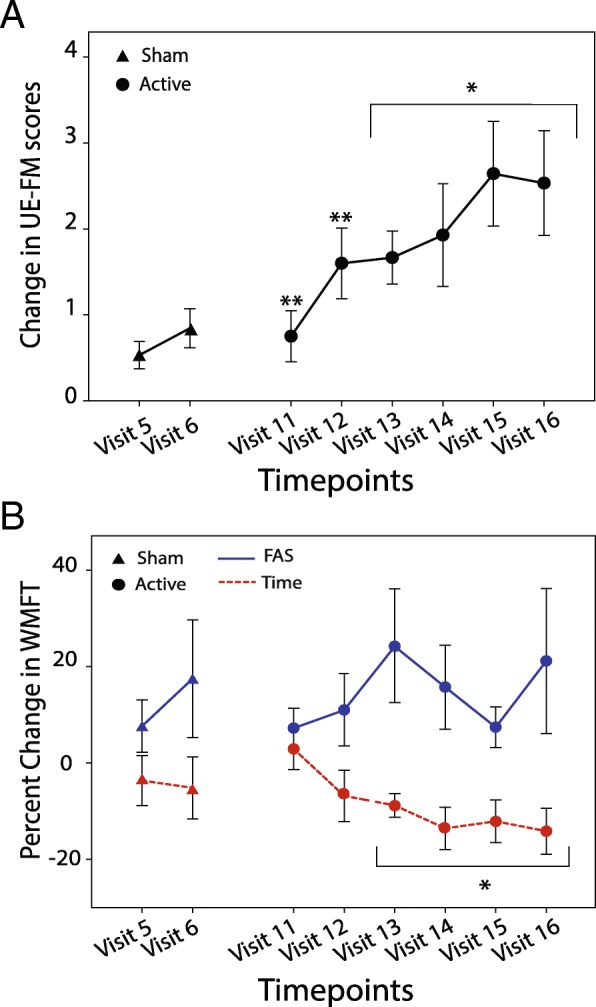
tsDCS+pDCS significantly improved Upper-Extremity Fugl-Meyer and Wolf Motor Function Test. **a** UE-FM score change (mean +/− SEM) immediately following 5 days of sham or treatment (visit 5 and 11; **P < 0.00625, Wilcoxon); and one week later (visits 6,12; **P < 0.00625, Wilcoxon). For the follow-up, UE-FM (mean+/−SEM) scores improved significantly from visits 12–16 (*P < 0.00625, Wilcoxon, visit 6 control). **b** WMFT-FAS score and WMFT Time score change (mean +/− SEM) immediately following 5 days of sham or treatment (visit 5 and 11; NS, Wilcoxon); and one week later (visits 6,12; NS, Wilcoxon). For the follow-up, WMFT Time scores (mean+/−SEM) improved significantly from visits 13–16 (*P < 0.00625, Wilcoxon, visit 6 control); WMFT-FAS scores one to five weeks later (NS, Wilcoxon)

Secondary outcomes, UE-FM and WMFT Test, were performed to evaluate functional improvements after each treatment condition (Fig. 4a and b). For these two measures, only patients who had completed both the sham and active treatment phases and at least 3 active treatment follow-up evaluations were included in the analysis (*N* = 16). For the UE-FM there were no significant differences between conditions immediately after treatment, between visit 5 and visit 11, however a significant difference between conditions was observed at visit 6 compared to visit 12 (*P* = .005; Wilcoxon). UE-FM measures increased significantly from visit 11–16 (*P* < .005; Wilcoxon). Using the clinically established MCID (a change in score of greater than 4.00–4.25), the responder rate was 37.50% (6/16). For the WMFT, there were no significant differences between the two conditions at visit 5 and 11 for either Time or FAS. However, over time there was a significant difference in WMFT-Time at visit 13 through 16 (*P* < .004, Wilcoxon). FAS scores improved but not significantly. Raw scores for UE-FM and WMFT are presented in Table S2 and S3 respectively.

### Clinical test of blinding strategy

Upon study completion 66% (14/21) of the patients correctly identified sham and active treatment conditions; however only 14% (2/14) were completely sure of their choice and 86% (12/14) were offering educated or blind guesses. The remaining patients, 33% (7/21), could not discriminate sham from treatment condition. In view of the uncertainty in most of the patients about the discrimination of sham and treatment condition, we viewed the blinding as effective.

## Discussion

This first clinical trial of paired tsDCS+pDCS in patients with chronic post-stroke hemiparesis demonstrated that five consecutive 20-min sessions of active anodal stimulation significantly reduced upper limb spasticity for up to 5 weeks after intervention and was associated with significant improvements in motor function. The improvements in the Modified Tardieu Scale score summed across the upper extremity were significant at the end of active treatment and for five weeks of follow-up measures, which was consistent with the improvements captured by objective measures. While the MTS score for the wrist flexor alone did not reach the significance, it trended toward a reduction in spasticity. Of note, there were no adverse effects during this study. These positive effects of the active paired tsDCS+pDCS treatments on spasticity occurred in subjects with chronic (on average 3 years) stroke and who had a wide range of functional severity levels (mean admission Upper Extremity Fugl-Meyer score equals 27, indicating moderate upper limb impairment) so that the range 8–55 points included severe and mild upper limb motor impairment. In practice, patients with lower UE-FM scores might benefit from paired tsDCS+pDCS treatment in order that they not be prematurely excluded from additional treatment studies.

After acute stroke most patients who enter an in-patient facility will undergo a variety of re-training protocols, some more intense than others. The motor outcome after 2–6 weeks of these programs results generally in most subjects walking independently, often with a device or orthosis, but many fewer subjects regain upper extremity motor function (Hendricks et al., 2002; Kwakkel et al., 2003; Shelton & Reding, 2001). Our results unexpectedly showed that patients treated with an anti-spasticity regimen alone, and without the standard prescribed activity-dependent training, demonstrated significant improvements in motor function, and, in fact, reflect the anecdotal reports from patients of modestly improved motor behaviors. Although the motor changes for the group did not reach an MCID, the responder rate of 37.50% and the 5 week durability should contribute to future study designs and perhaps new approaches to motor recovery therapy.

In clinical settings, the current standard of care for spasticity remains recurrent botulinum toxin injections. This neuromuscular blocking agent alters spasticity by blocking the release of acetylcholine presynaptically at the neuromuscular junction thereby reducing the involuntary activation of the spastic muscle (Li, 2017). While there is a consensus regarding the ability of botulinum toxin to transiently reduce spasticity and muscle stiffness (Li, 2017; Gracies et al., 2015; Smith et al., 2000; Bakheit et al., 2000; Simpson et al., 1996; Devier et al., 2017), this mechanism causes muscle weakness, and likely limits additional functional recovery (Hodics et al., 2012; Devier et al., 2017). A recent study reports that botulinum toxin injections paired with rehabilitation significantly decreased spasticity and improved motor functions, whereas botulinum toxin treatment alone did not lead to functional improvements (Devier et al., 2017).

In contrast to the results from botulinum toxin studies, in the present study, some motor improvements were observed as early as one week after treatment, and were maintained for five weeks, in absence of any additional training. The improved motor performance following paired tsDCS+pDCS may be attributed to different mechanisms of actions compared to botulinum toxin. While the latter acts by blocking the release of acetylcholine at the neuromuscular junction, tsDCS+pDCS likely acts by reducing the hyperexcitability of spinal motor neurons (Mekhael et al., 2019), a result thought to be dependent on decreased cortical inhibition after stroke (Katz & Zev Rymer, 1989; D'Amico et al., 2014; Wissel et al., 2013; Lee et al., 2014). More specifically, recent preclinical data suggest molecular targets involved in a candidate mechanism for spinal hyperexcitability. These experiments show that following neurological insult there is an imbalance in protein levels of sodium potassium chloride cotransporter isoform 1 (NKCC1) and potassium chloride cotransporter isoform 2 (KCC2). Both proteins are involved in establishing chloride gradients across the neuronal membrane (Mekhael et al., 2019; Boulenguez et al., 2010). Moreover, paired tsDCS+pDCS in spastic SCI mice demonstrated downregulation of NKCC1 expression in spinal motor neurons that was accompanied by significant reduction in spasticity as well as improved locomotion (Boulenguez et al., 2010). Reduction of NKCC1 through either pharmacological blockade or non-invasive stimulation using paired tsDCS+pDCS resulted in decreased levels of intracellular [Cl-] and therefore, enhanced GABA-ergic presynaptic inhibition (Boulenguez et al., 2010). These results in mice with spinal cord injury clearly have implications for the effect of paired tsDCS+pDCS for patients with stroke.

Post-stroke spasticity that occurs after injury to the upper motor neuron, is also accompanied by a constellation of positive and negative symptoms including muscle weakness, loss of dexterity, impaired motor control, and reduced endurance (Sommerfeld et al., 2012; Mekhael et al., 2019; D'Amico et al., 2014). These symptoms, which constitute upper-motor neuron syndrome have been attributed to alpha motoneurons hyperexcitability (Mekhael et al., 2019; Katz & Zev Rymer, 1989). Combining novel pre-clinical experiments with these positive clinical results prompts a mechanistic hypothesis that paired tsDCS and pDCS ameliorates the effects of an upper motor neuron syndrome. Anecdotal reports from patients in this study suggest that the changes registered in decreased spasticity on the subjective and objective measures were apparent to them as their most frequent comment was about the regained ability to lay their affected hand flat on a table and to feel their arm “definitely looser”. The fact that spasticity reduction and motor improvements are sustained over a period of weeks following intervention strongly supports the possibility of achieving greater functional improvements with the introduction of motor training during this period.

## Conclusion

The current data show that bioelectronic approaches to clinical problems may generate novel outcomes. The present study demonstrates the potential of paired tsDCS+pDCS to reduce deleterious motor symptoms associated with lesions to the upper motor neuron after stroke and improve functional recovery in the absence of direct motor training. A pivotal, double-blind, multi-center clinical trial is needed to further establish the effectiveness of this. Additional studies to investigate whether the critical cellular and molecular mechanisms in humans are comparable to the pre-clinical experiments are also warranted.

## Data Availability

The data that support the findings of this study are available from Johanna L Chang, Senior Research Coordinator. Data are available from the authors upon reasonable request and with permission of PathMaker Neurosystems, Inc.

## Abbreviations

CNS: Central nervous system
CT: Computerized tomography
mA: milliamperes
MAS: Modified Ashworth Scale
MCID: Minimal clinically important improvement
MDC: Minimum detectable change
MRI: Magnetic resonance imaging
MTS: Modified Tardieu Scale
MTS_flexor_: Modified Tardieu scale for wrist flexor
MTS_UE_: Modified Tardieu Scale for the upper extremity muscles, including shoulder: horizontal adductors, vertical adductors, internal rotators; elbow: flexors, extensors, pronators, supinators; wrist/hand: flexors, extensors, fingers and palmar interossei+ flexor digitorum superficialis
pDCS: peripheral nerve direct current stimulation
ROM: Range of motion
SD: Standard deviation
SEM: Standard error of the mean
tsDCS: trans-spinal direct current stimulation
UE-FM: Upper extremity Fugl-Meyer scale
V1: Velocity slow, slow speed
V2: Velocity fast, fast speed
WMFT: Wolf Motor Function test
WMFT-FAS: functional ability scale qualitative score for the WMFT
WMFT-Time: timed performance of the WMFT
